# Distinct Behaviour of Sorafenib in Experimental Cachexia-Inducing Tumours: The Role of STAT3

**DOI:** 10.1371/journal.pone.0113931

**Published:** 2014-12-01

**Authors:** Míriam Toledo, Fabio Penna, Sílvia Busquets, Francisco J. López-Soriano, Josep M. Argilés

**Affiliations:** 1 Cancer Research Group, Departament de Bioquímica i Biologia Molecular, Facultat de Biologia, Universitat de Barcelona, Barcelona, Spain; 2 Institut de Biomedicina de la Universitat de Barcelona, Barcelona, Spain; 3 Department of Clinical and Biological Sciences, University of Torino, Turin, Italy; City of Hope, United States of America

## Abstract

The presence of a tumour is very often associated with wasting in the host, affecting both skeletal muscle and adipose tissue. In the present study we used sorafenib, a multi-kinase inhibitor with anti-tumour activity, in order to investigate the effects of chemotherapy on wasting. Three different experimental mouse tumour models were included: C26 colon carcinoma, B16 melanoma and Lewis lung carcinoma (LLC). The results obtained clearly show that sorafenib was effective in reducing tumour growth in LLC and B16 models, while it had no effect on C26. Interestingly, sorafenib treatment reduced the signs of muscle wasting and improved the physical activity in the LLC model and also in the C26, despite the absence of antineoplastic action in the latter. Our results discard a role for IL-6 in the action of sorafenib since the drug did not affect the levels of this cytokine. Conversely, sorafenib seems to act by influencing both STAT3 and ERK activity at muscle level, leading to reduced accumulation of Pax7 and atrogin-1. Sorafenib may interfere with muscle wasting by decreasing the activation of these signal transduction pathways.

## Introduction

Development of cachexia is often found in cancer patients. It occurs in 50 to 80% of these patients [1], and is considered as a predictor of reduced survival accounting for more than 20% of cancer patients deaths [2]. It reduces physical activity [3] and quality of life and diminishes the tolerance to anticancer therapy [4], [5]. Cachexia is defined, according to an international consensus [6], as a “complex metabolic syndrome associated with underlying illness and characterized by loss of muscle with or without loss of fat mass. The prominent clinical feature of cachexia is weight loss in adults (corrected for fluid retention) or growth failure in children (excluding endocrine disorders). Anorexia, inflammation, insulin resistance and increased muscle protein breakdown are frequently associated with cachexia. Cachexia is distinct from starvation, age-related loss of muscle mass, primary depression, malabsorption and hyperthyroidism and is associated with increased morbidity”. The metabolic disturbances found in the cancer patient include an increased energy inefficiency, insulin resistance and altered carbohydrate metabolism, adipose tissue dissolution and hypertriglyceridemia and muscle wasting. All of these alterations have a causative role in the development of the cachexia syndrome [7],[8].

The loss of body weight results from both decreased adipose tissue and muscle; however, muscle wasting should be regarded as the most important since it plays a key role in recovery from cancer cachexia. Thus, it is an important determinant of survival and muscle strength and function, central to the recovery process [9]. Muscle wasting occurs when synthesis is decreased, breakdown is increased, or a combination of events leads to a net negative balance. The regulation of skeletal muscle is managed by several transcription factors and intracellular signalling pathways (anabolic or catabolic), and a chronic imbalance in the activation of these different pathways leads to muscle wasting [10]–[12]. Although there are several mediators involved in the metabolic alterations linked to muscle wasting, pro-inflammatory cytokines seem to play a major role [13].

Many efforts have been involved in designing a treatment for the cachectic syndrome, but unfortunately there is not a single one fully satisfactory in reversing weight loss. The development of different therapeutic strategies has focused on two targets: counteracting anorexia and neutralizing metabolic disturbances [14]. However, providing complete nutritional requirements by way of total parenteral nutrition does not abrogate weight loss [8]. Instead, many drugs have been proposed and used in clinical trials [15], [16], while others are still under investigation using experimental animals in order to revert metabolic alterations [17], [18]. However, there are no effective treatments actually adopted in the clinical practice.

During the last decades several experimental models were established to study cancer cachexia [19]. However, tumour-injected animals as substitute for cancer patients have a major drawback in the omission of anti-tumour treatments that frequently complicate patient management. Such treatments might improve, do not affect, or even worsen cachexia, according to their effectiveness in getting rid of the tumour or to secondary adverse effects.

New focused cancer treatments are less toxic than traditional chemotherapy. Such therapies ought to make tumour cells more susceptible, without increasing host toxicity. From this point of view, sorafenib is a multikinase inhibitor that has shown efficacy against a wide variety of tumours in preclinical models [20] and it has been approved in humans for the treatment of advanced hepatocellular carcinoma [21] and advanced renal cell carcinoma[22]. Nevertheless, Antoun et al. [23] reported that long term administration of sorafenib is associated with muscle wasting in patients with advanced renal cell carcinoma. Recent studies showed that it inhibits cell proliferation by targeting the Raf/MEK/ERK signalling pathways and exerts an anti-angiogenic effect by inhibition of tumour angiogenesis through VEGFR and PDGFR [24], [25]. Recently Yang et al. [26] reported that sorafenib treatment blocked the IL-6-dependent STAT3 phosphorylation in cancer cells. Such effect is mediated by the inhibition of STAT3 up-stream kinases JAK1 and JAK2 [27]. IL-6 is a critical cytokine secreted in several cancer types, and circulating levels of this cytokine have been shown to correlate with weight loss in cancer patients and reduced survival [28], [29].

The aim of the present study was to characterize the cachectic phenotype of three commonly used experimental tumours in mice treated with sorafenib, in order to provide a reliable tool for translational research.

## Materials and Methods

### Experimental tumour models

C57BL/6 (for LLC and B16 tumour models) or Balb/C (for C26 tumour model) mice weighing about 20 g (Harlan, Barcelona, Spain) were maintained on a regular dark-light cycle (light from 08:00 to 20:00), with free access to food and water during the whole experimental period. They were cared for in compliance with the *Policy on Humane Care and Use of Laboratory Animals* (ILAR 2011). The Bioethical Committee of the University of Barcelona approved the experimental protocol. All animal manipulations were made in accordance with the European Community guidelines for the use of laboratory animals.

The mice were randomized and divided into two groups, namely controls (C, n = 6) and tumour bearers (TB). TB mice were inoculated alternatively subcutaneously in the back with 5×10^5^ B16 or C26 cells or i.m. (hind leg) with 5×10^5^ LLC cells (see below for cell expansion). TB animals were divided into two sub-groups: untreated (n = 8) and treated (intragastric administration) every day with sorafenib (90 mg/kg of initial body weight; n = 8). Mice were sacrificed under anaesthesia 14 days after tumour implantation. The day of sacrifice, the animals were weighed and anaesthetized with an intraperitoneal injection of ketamine/xylazine mixture (3∶1; Imalgene® and Rompun® respectively). Several tissues and the tumour were rapidly excised, weighed, and frozen in liquid nitrogen.

Colon26 carcinoma (C26) and B16 melanoma (B16) cells were maintained *in vitro* in Dulbecco's Modified Eagle's Medium (DMEM, Invitrogen) supplemented with 10% FBS, 100 U/mL penicillin, 100 µg/mL streptomycin, 100 µg/mL sodium pyruvate, 2 mM L-glutamine, at 37°C in a humidified atmosphere of 5% CO_2_ in air. The day of tumour implantation the cells were trypsinized, re-suspended in sterile saline and rapidly injected in the mice. Lewis lung carcinoma (LLC) cells were obtained from previous tumour hosts harvesting the cells in the exponential growth phase. The LLC is a cachexia-inducing, rapidly growing murine metastatic tumour composed by poorly differentiated cells, with a relatively short doubling time. The number of lung metastases was evaluated by light microscopy after necropsy. The metastases weight was evaluated according the methodology used by Donati et al [30].

### Haematocrit

Total blood was withdrawn from anaesthetized mice by cardiac puncture and collected in heparinized tubes. A drop was used to fill haematocrit capillary tubes that were centrifuged in a haematocrit centrifuge for 5 min at 800 x g. Haematocrit was calculated as percentage of packed cell volume of the total blood.

### Total physical activity

Total physical activity (IR ACTIMETER System and ACTITRAK software from Panlab, Barcelona) was determined during the last 24 hours before the sacrifice of the animals in control mice and tumour-bearing mice (both non-treated and treated with sorafenib), using activity sensors which translate individual changes in the infrared pattern caused by movements of the animals into arbitrary activity counts. For the measurements, animals remained in their home cage. A frame containing an infrared beam system was placed on the outside of the cage; this minimised the stress of the animals.

### ELISA

IL-6 serum levels were detected by a commercially available mouse ELISA kit, used according to the manufacturer instructions (Diaclone, bioNova, Spain). Serum from each animal (50 µL) was assayed in duplicate. Quantitative calibration was obtained performing a standard curve with recombinant mouse IL-6.

### Western Blotting

50 mg of gastrocnemius muscle were homogenized in 10 mM HEPES, pH 7.5, containing 10 mM MgCl_2_, 5 mM KCl, 0.1 mM EDTA, pH 8.0, 0.1% Triton X-100, with freshly added protease and phosphatase inhibitors. Samples were then centrifuged (5 min, 3000 g), the supernatant collected as the cytosolic fraction and the pellet resuspended in 20 mM HEPES, pH 7.9, containing 25% glycerol, 500 mM NaCl, 1.5 mM MgCl_2_, 0.2 mM EDTA, pH 8.0, protease and phosphatase inhibitor and incubated on ice for 30 min. Cell debris were removed by centrifugation (5 min, 3000 g) and the supernatant (nuclear fraction) collected. Protein concentration was assayed using BSA as working standard. Equal amounts of protein (30 µg) were heat-denaturised in sample-loading buffer (50 mM Tris-HCl, pH 6.8, 100 mM DTT, 2% SDS, 0.1% bromophenol blue, 10% glycerol), resolved by SDS-PAGE and transferred to nitrocellulose membranes. The filters were blocked with Tris-buffered saline (TBS) containing 0.05% Tween and 5% non-fat dry milk and then incubated overnight with antibodies directed against: p-ERK (Tyr204), total-ERK, total-STAT3 and GAPDH (Santa Cruz Biotechnology, USA), FoxO3a and p-STAT3 (Tyr705; Cell Signalling Technology, Danvers, MA, USA), Atrogin-1 (ECM Biosciences, Versailles, KY) and the monoclonal antibody against Pax7 developed by Atsushi Kawakami, obtained from the Developmental Studies Hybridoma Bank (University of Iowa). Peroxidase-conjugated IgG (Bio-Rad, Hercules, USA) was used as secondary antibody. Membrane-bound immune complexes were detected by an enhanced chemiluminescence system (Santa Cruz Biotechnology, USA) on a photon-sensitive film (Hyperfilm ECL, GE Healthcare). Protein loading was normalized according to GAPDH expression. Quantification of the bands was performed by densitometric analysis using the ImageJ software.

### Statistical analysis

Statistical analysis of the data was performed by means of one-way analysis of variance (ANOVA). Statistically significant differences was analysed by post hoc Duncan test. All statistical tests were performed using SPSS version 21.

## Results and Discussion

**Figure 1:**
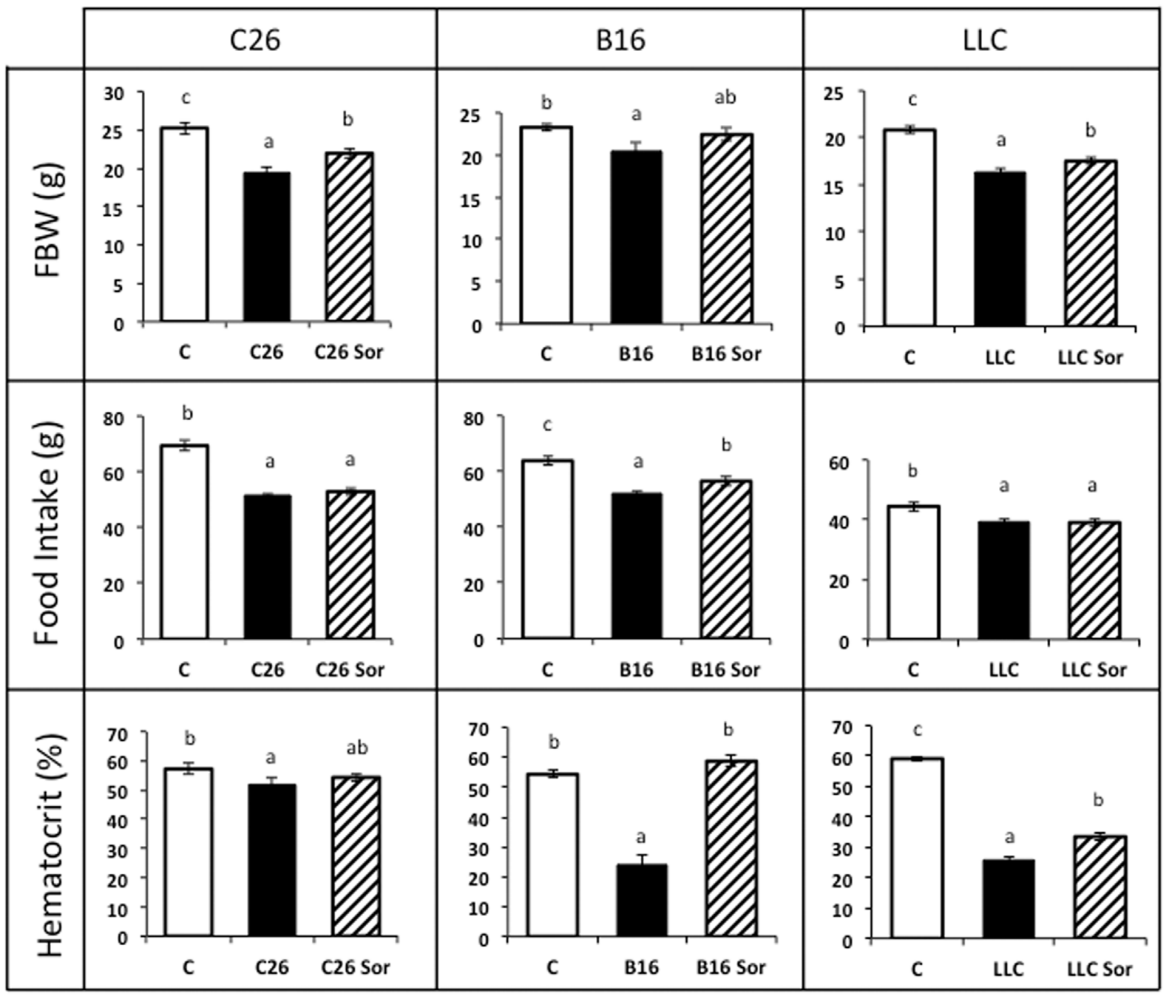
Body weight, food intake and haematocrit content in sorafenib-treated tumour-bearing mice. Results are mean ± S.E.M. for 6–8 animals. Food intake represents the total ingestion (g) during the period of the experiment prior to sacrifice, which took place 14 days after tumour inoculation. Final body weight excludes the tumour weight. The haematocrit is expressed in percentage. C: animals without tumour (n = 6); C26: C26 adenocarcinoma-bearing mice (n = 8); C26 Sor: C26 adenocarcinoma-bearing mice treated with sorafenib (n = 8); B16: B16 melanoma-bearing mice (n = 8); B16 Sor: B16 melanoma-bearing mice treated with sorafenib (n = 8); LLC: Lewis lung carcinoma-bearing mice (n = 8); LLC Sor: Lewis lung carcinoma-bearing mice treated with sorafenib (n = 8). Values that are significantly different of the results by one-way analysis of variance (ANOVA), statistically significant differences by post hoc Duncan test. Different letters in superscript indicate significant differences between groups.

In order to ascertain the effects of sorafenib on tumour-bearing animals we used three distinct experimental models: C26 colon carcinoma, B16 melanoma and Lewis lung carcinoma (LLC). All of them are associated with a clear cachectic response. From this point of view, as can be seen in Figure 1, body weight was reduced in all models as a consequence of tumour burden (23% C26, 12% B16, 24% LLC). Similar to the human condition, the reduction of body weight was associated with hypophagia in all the experimental models (Figure 1).

**Figure 2:**
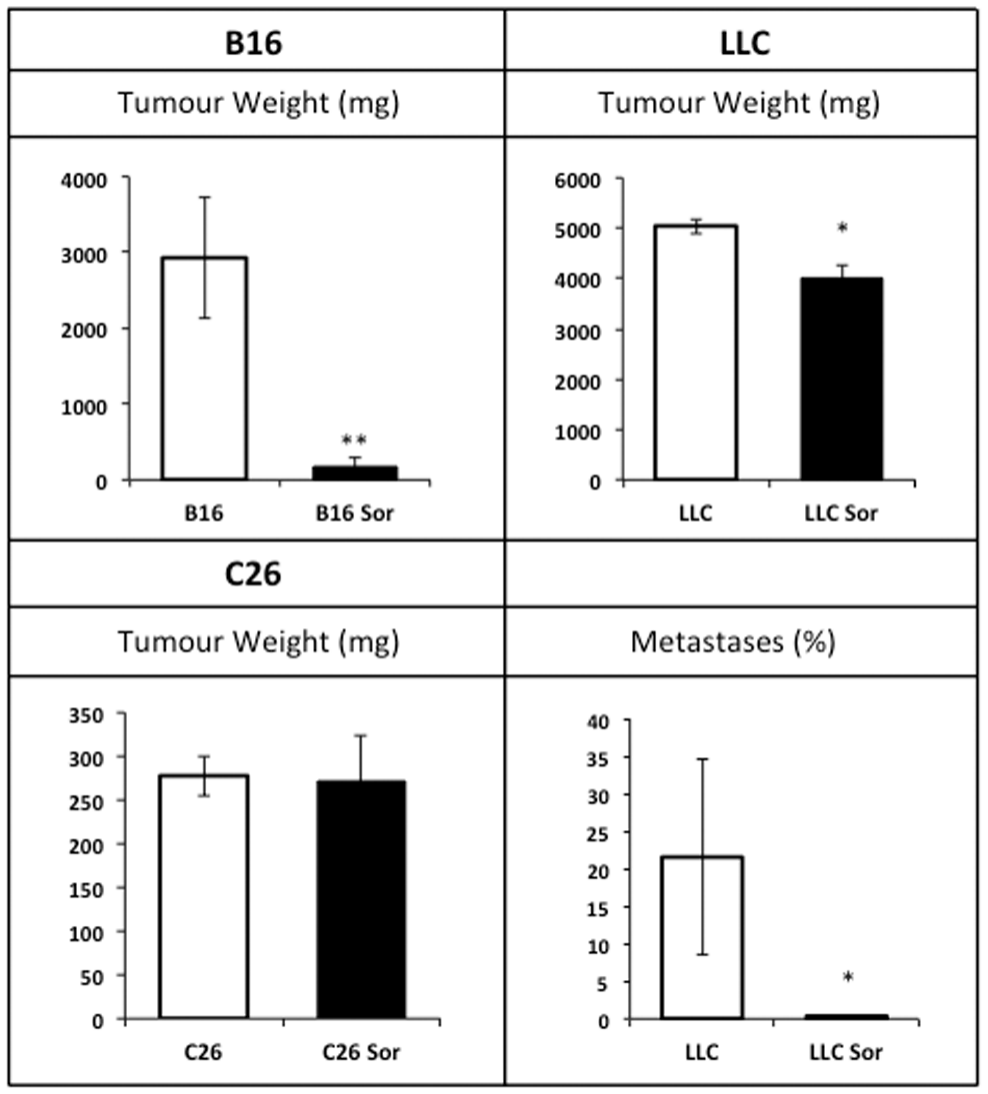
Tumour weights in sorafenib-treated mice. Results are mean ± S.E.M. for 6–8 animals. Tumour weights are expressed in g. In the LLC model metastatic content is represented as % of invaded lung surface. C26: C26 adenocarcinoma-bearing mice (n = 8); C26 Sor: C26 adenocarcinoma-bearing mice treated with sorafenib (n = 8); B16: B16 melanoma-bearing mice (n = 8); B16 Sor: B16 melanoma-bearing mice treated with sorafenib (n = 8); LLC: Lewis lung carcinoma-bearing mice (n = 8); LLC Sor: Lewis lung carcinoma-bearing mice treated with sorafenib (n = 8). Values that are significantly different of the results by one-way analysis of variance (ANOVA), * p<0.05; ** p<0.01.

Treatment with the anti-tumour sorafenib-inhibitor of the MAPK cascade- resulted in a huge reduction in tumour mass in the B16-bearing mice (94%) while it exerted only a small effect in the LLC (20% reduction of primary tumour mass and 98% fall of metastases number) (Figure 2). Remarkably, sorafenib had no effect on C26 tumour growth (Figure 2). The anti-tumoural treatment was able to markedly recover body weight in B16-bearing animals and also resulted in significant increases in body weight in C26 and LLC models although at a lesser extent than in the B16 model (Figure 1).

In the B16 model, sorafenib treatment increased food intake in comparison with the non-treated B16-bearing mice (Figure 1). Conversely, sorafenib treatment did not affect food intake in both the C26 and LLC models (Figure 1), suggesting that feeding behaviour mainly depends on the tumour burden rather than sorafenib administration.

Tumour growth led to a decrease in haematocrit in all the models tested, being especially marked in the B16 (56%) and the LLC (56%) mice, where the tumour mass was bigger (Figure 1). It is important to note that sorafenib administration was effective in increasing the haematocrit (as compared with the non-treated animals) in both the B16 and LLC models, proportionally to the reduction of tumour mass, thus linking directly the onset of anaemia with tumour burden. (Figure S1)

The decreased body weight was associated with a concomitant loss of muscle mass. As can be seen in Table 1, tumour growth promoted decreases in individual muscle weights in the three different models tested. In the case of C26, gastrocnemius (21%), tibialis (22%) and heart (13%) were decreased as a consequence of tumour growth. In the other models gastrocnemius and tibialis were significantly reduced (18% GSN, 14% tibialis for B16; 29% GSN, 27% tibialis for LLC). No effects of tumour growth were observed in the heart neither in the B16 nor in the LLC tumour models. Sorafenib treatment had significant effects in the rescue of both gastrocnemius and tibialis mass in the C26 animal model (Table 1), despite the ineffectiveness against tumour growth. Similarly, the treatment caused a recovery of the gastrocnemius muscle in the LLC model; however it did not have any statistical effects on the tibialis muscle. Concerning the muscle, in contrast with the prevention of body weight loss, no statistically significant effects were observed in the B16 animals after sorafenib administration.

**Table 1:** Muscle and heart weights in sorafenib-treated tumour-bearing mice.

Tumour model	Muscle	Experimental groups			
		C	T	T Sor	ANOVA
**C26**	GSN	562±9 ^c^	445±17 ^a^	500±15 ^b^	0,000
	Tibialis	192±3 ^c^	149±7 ^a^	168±6 ^b^	0,000
	Heart	502±15 ^b^	439±14 ^a^	477±16 ^ab^	0,025
**B16**	GSN	634±21 ^b^	522±24 ^a^	565±17 ^a^	0,010
	Tibialis	193±8 ^b^	166±8 ^a^	178±8 ^ab^	0,073
	Heart	586±31	514±21	512±25	ns
**LLC**	GSN	551±16 ^c^	390±18 ^a^	468±7 ^b^	0,000
	Tibialis	189±9 ^b^	138±6 ^a^	157±11 ^a^	0,005
	Heart	550±35	536±27	551±11	ns

Results are mean ± S.E.M. Muscle weights are expressed as mg/100 g of initial body weight. GSN: gastrocnemius muscle. C: animals without tumour (n = 6); T: tumour-bearing animals (n = 8); T Sor: tumour-bearing animals treated with sorafenib (n = 8). Values significantly different of the results by one-way analysis of variance (ANOVA), statistically significant differences by post hoc Duncan test. Different letters in superscript indicate significant differences between groups.

As can be seen in Table 2, in addition to skeletal muscle, tumour growth determined a very important decrease in white adipose tissue mass. Indeed, cachexia is linked with muscle wasting and adipose tissue loss [6], [31]. A recent report suggests that muscle wasting is linked to the loss of adipose tissue; if fat mobilization is prevented, muscle wasting does not take place, at least in experimental animals [32]. The largest reduction is observed in the LLC-bearing animals, with losses over 90% of total tissue. Sorafenib treatment induced a rescue of adipose tissues in all the cachexia models tested, the amelioration being especially marked in the case of B16 where almost total recovery is accomplished, explaining partially the above reported discrepancy between body and muscle wasting. This observation is particularly interesting since sorafenib, despite inducing an almost complete adipose tissue recovery, exerted only negligible effects on skeletal muscle. From this point of view, the present data do not support the conclusions of Das et al. [32], since the complete recovery of adipose tissue is not sufficient to promote muscle sparing.

**Table 2:** White adipose weights in sorafenib-treated tumour-bearing mice.

Tumour model	Adipose	Experimental groups			
		C	T	T Sor	ANOVA
**C26**	WATd	684±71 ^b^	29±18 ^a^	285±56 ^b^	0,000
	WATe	2043±172 ^b^	460±129 ^a^	1410±149 ^b^	0,000
**B16**	WATd	374±38 ^b^	140±29 ^a^	290±13 ^b^	0,000
	WATe	1554±103 ^b^	811±73 ^a^	1425±24 ^b^	0,000
**LLC**	WATd	315±47 ^b^	16±3 ^a^	35±4 ^a^	0,000
	WATe	1298±9 ^b^	171±36 ^a^	259±14 ^a^	0,000

Results are mean ± S.E.M. Adipose weights are expressed as mg/100 g of initial body weight. WATd: White adipose tissue dorsal; WATe: White adipose tissue epididymal. C: animals without tumour (n = 6); T: tumour-bearing animals (n = 8); T Sor: tumour-bearing animals treated with sorafenib (n = 8). Values significantly different of the results by one-way analysis of variance (ANOVA), statistically significant differences by post hoc Duncan test. Different letters in superscript indicate significant differences between groups.

**Figure 3:**
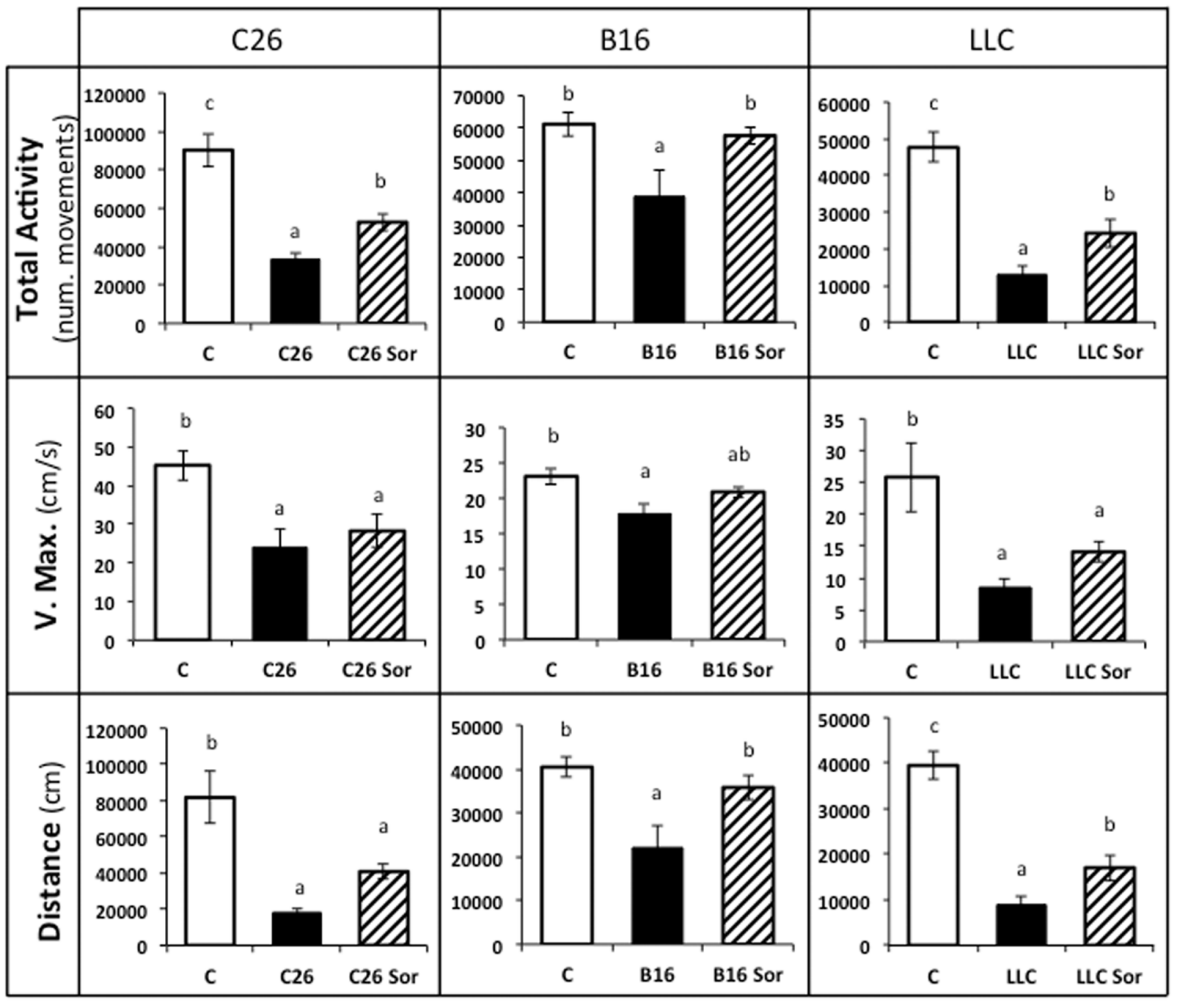
Kinetic parameters in sorafenib-treated tumour-bearing mice. Results are mean ± S.E.M. for 6–8 animals. Physical activity is expressed in number of movements. Maximum velocity is expressed in cm/s. Travelled distance is expressed in cm. C: animals without tumour (n = 6); C26: C26 adenocarcinoma-bearing mice (n = 8); C26 Sor: C26 adenocarcinoma-bearing mice treated with sorafenib (n = 8); B16: B16 melanoma-bearing mice (n = 8); B16 Sor: B16 melanoma-bearing mice treated with sorafenib (n = 8); LLC: Lewis lung carcinoma-bearing mice (n = 8); LLC Sor: Lewis lung carcinoma-bearing mice treated with sorafenib (n = 8). Values that are significantly different of the results by one-way analysis of variance (ANOVA), statistically significant differences by post hoc Duncan test. Different letters in superscript indicate significant differences between groups.

Taking into consideration the fact that the rescue of muscle mass does not necessarily mean a concomitant recovery of muscle function [33], we decided to measure total physical activity as a surrogate for muscle function and behavioural adaptation. Figure 3 depicts total activity, maximum speed and distance travelled by the animals. As it can be seen, tumour burden resulted in a considerably decrease in all these parameters. The most affected animals were the LLC mice, where total activity (78%), maximum velocity (65%), and distance (82%) were reduced. Sorafenib treatment improved total physical activity in the three models studied. It is interesting to remark that the largest improvement was observed in the B16 model, precisely where sorafenib treatment was not able to fully revert muscle wasting. Therefore, some other factors must be taken into consideration for explaining the improvement in total activity, including the reduction of the tumour mass. It is possible to speculate that animal activity does not rely on the muscle mass alone, rather depending also on the energy availability (fat included) and the presence of anaemia, a clear limiting factor for physical activity. Sorafenib treatment also improved maximum velocity in the LLC model (Figure 3). Concerning the distance travelled, the treatment improved this parameter in all the models tested, independently from the distinct effect exerted on tumour burden.

**Figure 4:**
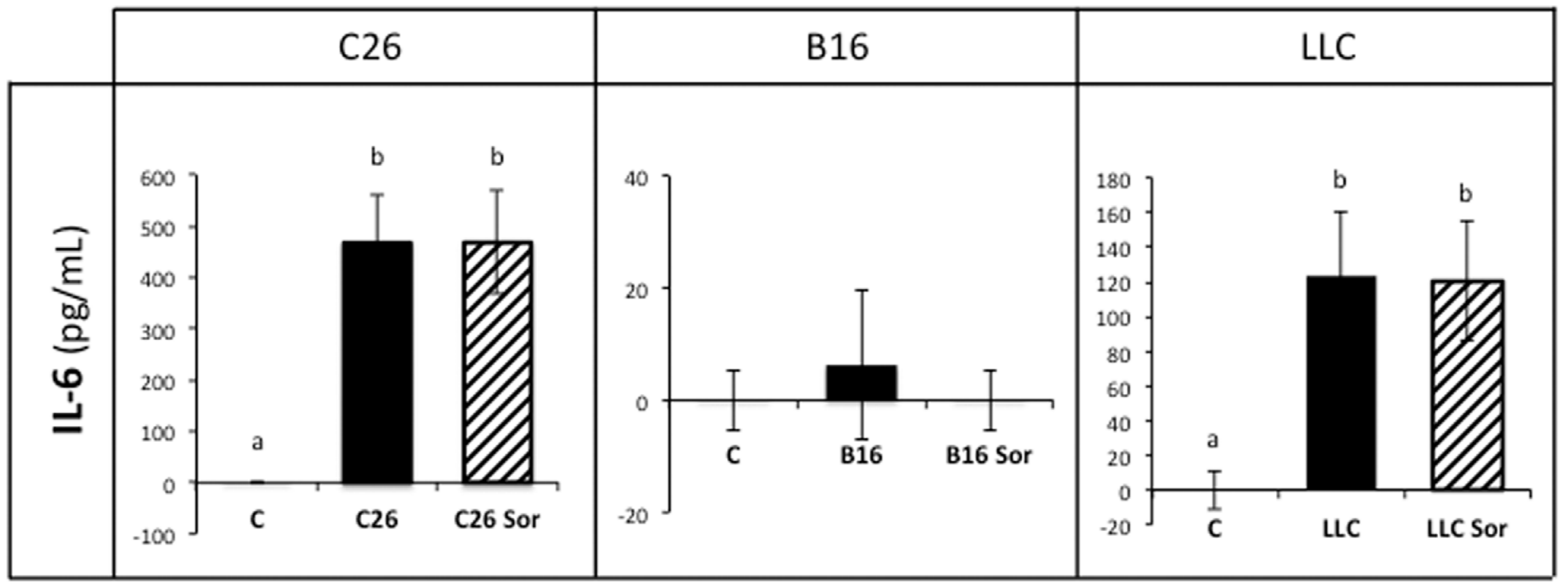
Circulating IL-6 levels in sorafenib-treated tumour-bearing mice. Results are mean ± S.E.M. for 6–8 animals. IL-6 levels are expressed in pg/mL. C: animals without tumour (n = 6); C26: C26 adenocarcinoma-bearing mice (n = 8); C26 Sor: C26 adenocarcinoma-bearing mice treated with sorafenib (n = 8); B16: B16 melanoma-bearing mice (n = 8); B16 Sor: B16 melanoma-bearing mice treated with sorafenib (n = 8); LLC: Lewis lung carcinoma-bearing mice (n = 8); LLC Sor: Lewis lung carcinoma-bearing mice treated with sorafenib (n = 8). Values that are significantly different of the results by one-way analysis of variance (ANOVA), statistically significant differences by post hoc Duncan test. Different letters in superscript indicate significant differences between groups.

**Figure 5:**
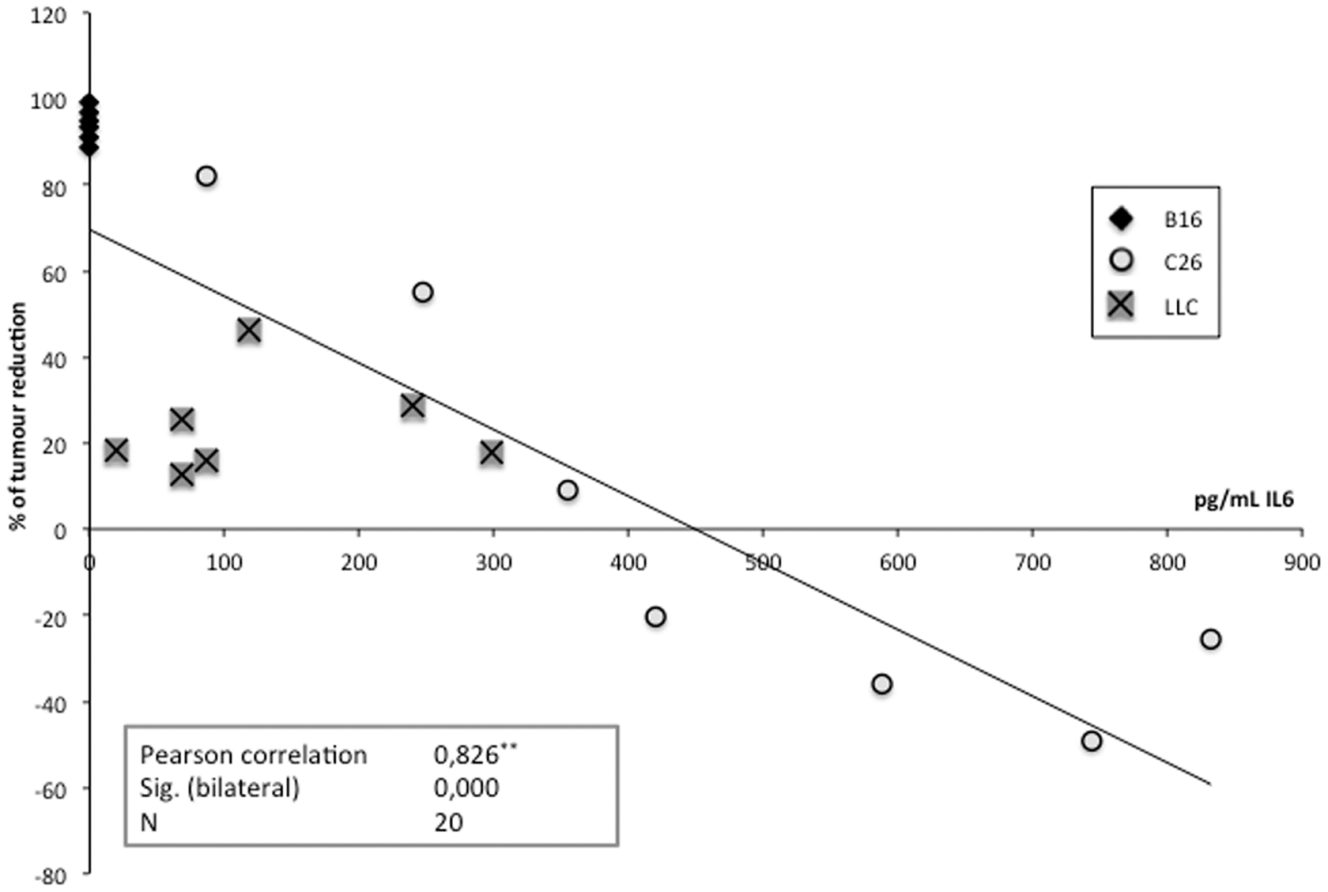
Correlation between the tumour reduction induced by sorafenib and the circulating IL-6 levels. N = 20 (B16, n = 6; C26, n = 7; LLC, n = 7). Pearson Correlation. ** Correlation is statistically significant at 0,01 level (bilateral).

In order to explain the mechanisms accounting for the tumour-independent sorafenib effects, we determined the circulating concentrations of IL-6, a cytokine that has been reported to be affected by sorafenib, which reduces its release [26]. Interestingly, in both C26 and LLC-bearing mice, where the levels of IL-6 are high as a consequence of tumour growth, sorafenib did not lower the cytokine concentration, suggesting that sorafenib effects do not directly rely on IL-6 inhibition (Figure 4). It has to be pointed out that muscle IL-6 concentration could be altered by the treatment; differences in receptor content are another possibility. Unfortunately, we have not assessed these possibilities. However, it is interesting to point out that the anti-tumour effect of sorafenib was stronger when IL-6 circulating levels were low, as in the B16-bearing mice, existing a good correlation between the degree of tumour reduction and the concentration of IL-6 (Figure 5) in the different models tested. It seems, therefore, that sorafenib anti-tumour action is more effective in low IL-6 conditions.

**Figure 6:**
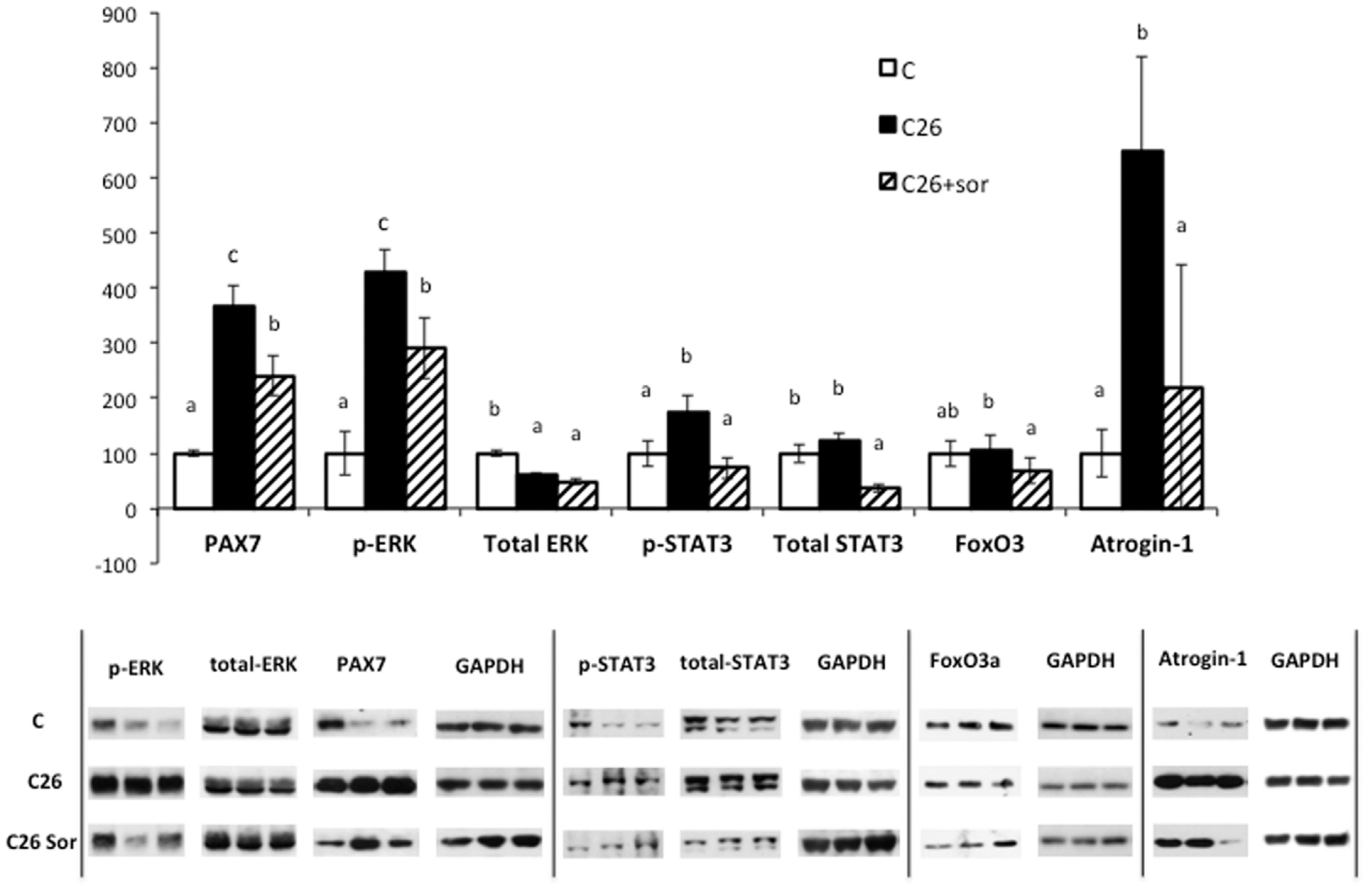
Intracellular signals of skeletal muscle (gastrocnemius) in sorafenib-treated mice bearing the C26 adenocarcinoma. Results are mean ± S.E.M. C: animals without tumour; C26: C26 carcinoma-bearing mice; C26+sor: C26 adenocarcinoma-bearing mice treated with sorafenib. Values that are significantly different of the results by one-way analysis of variance (ANOVA), statistically significant difference by post hoc Duncan test. Different letters in superscript indicate significant differences between groups.

**Figure 7:**
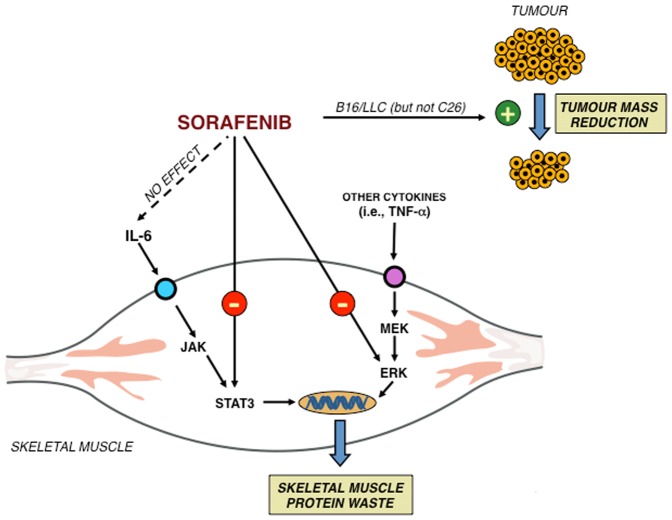
Possible intracellular pathways linked with sorafenib action in skeletal muscle.

In order to further investigate the mechanisms by which sorafenib exerts its effects, we determined the intramuscular concentration and activation state of several proteins that might be involved in the sorafenib action. As far as we are concerned, there are no reports suggesting a direct kinase-inhibiting action of sorafenib in the skeletal muscle. For this part of the study we only used the C26 model, since in this setting, despite the absence of drug effects on tumour burden, sorafenib improved both muscle weight and activity parameters. The results depicted in Figure 6 show that tumour growth resulted in a significant nuclear accumulation of Pax7, a protein involved in muscle regeneration [34], [35], concomitant with the hyperactivation of the kinases ERK and STAT3, that were reported to be critical for the onset of muscle wasting in C26 mice [34]–[36]. Surprisingly, sorafenib significantly decreased the concentration of Pax7 and the activation of the above-mentioned kinases. Consistent with previous observations showing that ERK inhibition prevented the activation of the ubiquitin ligase atrogin-1 [34], sorafenib administration was able to reduce the atrogin-1 protein accumulation in C26 mice (Figure 6), likely limiting the catabolic stimulus. Such effect seems independent from the activation of the transcription factor FoxO3a, as the nuclear (i.e. active) levels of the protein did not change in C26 mice and slightly decreased in sorafenib-treated C26 animals, confirming that the Akt/FoxO pathway is not involved in the onset of muscle wasting [37], at least in this experimental setting. These results suggest that sorafenib exerts its anti-cachectic action directly on the muscle by inhibiting either the STAT3 or ERK pathways, independently from IL-6 serum levels (Figure 7). Recent observations point out the suppressive role played by STAT3 in satellite cell-mediated muscle regeneration [38], [39] and, consistently, the anti-cachectic action exerted by sorafenib is stronger in the experimental model of cachexia where muscle regeneration is more affected.

In conclusion, the results presented here reinforce the idea that sorafenib is an efficient drug for the treatment of tumours in addition to being a candidate for anti-cachectic therapy. This is based on the fact that any reduction in tumour mass results in a reduction in cachexia and also in the fact that, even without affecting the tumour, it does behave as an anti-cachectic molecule, as observed in the C26 tumour model. Yet, previous studies actually conclude that sorafenib *per se* induces wasting [23], [40]; therefore further studies in this field are needed and might lead to a new promising drug combination for the treatment of cachexia.

## Supporting Information

Figure S1 **Correlation between tumour mass (expressed as % of the mean) and haematocrit in the three tumour models.** Pearson analysis shows a significant correlation of tumour versus haematocrit in B16 (Pearson r = 0.789; p = 0.004, n = 12) and a tendency in LLC (Pearson r = 0.526; p = 0.065, n = 13). No correlation is present in C26 (Pearson r = 0.120; p = 0.6, n = 14).
(TIF)

## References

[1] Muscaritoli M, Bossola M, Aversa Z, Bellantone R, Rossi Fanelli F (2006) Prevention and treatment of cancer cachexia: new insights into an old problem. Eur J Cancer 42:31–41 Available: http://www.ncbi.nlm.nih.gov/pubmed/16314085. 16314085 10.1016/j.ejca.2005.07.026

[2] Loberg RD, Bradley DA, Tomlins SA, Chinnaiyan AM, Pienta KJ. (n.d.) The lethal phenotype of cancer: the molecular basis of death due to malignancy. CA Cancer J Clin 57:225–241 Available: http://www.ncbi.nlm.nih.gov/pubmed/17626119. Accessed: 2013 Dec 15. 10.3322/canjclin.57.4.225 17626119

[3] Moses AWG, Slater C, Preston T, Barber MD, Fearon KCH (2004) Reduced total energy expenditure and physical activity in cachectic patients with pancreatic cancer can be modulated by an energy and protein dense oral supplement enriched with n-3 fatty acids. Br J Cancer 90:996–1002 Available: http://www.pubmedcentral.nih.gov/articlerender.fcgi?artid=2409623&tool=pmcentrez&rendertype=abstract. Accessed: 2013 Dec 15. 14997196 10.1038/sj.bjc.6601620 PMC2409623

[4] Dewys WD, Begg C, Lavin PT, Band PR, Bennett JM, et al (1980) Prognostic effect of weight loss prior to chemotherapy in cancer patients. Eastern Cooperative Oncology Group. Am J Med 69:491–497. 7424938 10.1016/s0149-2918(05)80001-3

[5] Muscaritoli M, Anker SD, Argilés J, Aversa Z, Bauer JM, et al (2010) Consensus definition of sarcopenia, cachexia and pre-cachexia: joint document elaborated by Special Interest Groups (SIG) “cachexia-anorexia in chronic wasting diseases” and “nutrition in geriatrics”. Clinical nutrition (Edinburgh, Scotland). Vol. 29 pp 154–159 doi:10.1016/j.clnu.2009.12.004 10.1016/j.clnu.2009.12.004 20060626

[6] Evans WJ, Morley JE, Argilés J, Bales C, Baracos V, et al (2008) Cachexia: a new definition. Clin Nutr 27:793–799 Available: http://www.ncbi.nlm.nih.gov/pubmed/18718696. Accessed: 2013 Nov 11. 18718696 10.1016/j.clnu.2008.06.013

[7] Argilés JM, Alvarez B, López-Soriano FJ (1997) The metabolic basis of cancer cachexia. Med Res Rev 17:477–498 Available: http://www.ncbi.nlm.nih.gov/pubmed/9276862. 9276862 10.1002/(sici)1098-1128(199709)17:5<477::aid-med3>3.0.co;2-r

[8] Ng EH, Lowry SF (1991) Nutritional support and cancer cachexia. Evolving concepts of mechanisms and adjunctive therapies. Hematol Oncol Clin North Am 5:161–184 Available: http://www.ncbi.nlm.nih.gov/pubmed/1902829. Accessed: 2013 Dec 15. 1902829

[9] Wolfe RR (2006) The underappreciated role of muscle in health and disease. Am J Clin Nutr 84:475–482 Available: http://www.ncbi.nlm.nih.gov/pubmed/16960159. 16960159 10.1093/ajcn/84.3.475

[10] Hasselgren PO, Fischer JE (2001) Muscle cachexia: current concepts of intracellular mechanisms and molecular regulation. Ann Surg 233:9–17 Available: http://www.pubmedcentral.nih.gov/articlerender.fcgi?artid=1421177&tool=pmcentrez&rendertype=abstract. Accessed: 2014 Mar 13 11141219 10.1097/00000658-200101000-00003 PMC1421177

[11] Skipworth RJE, Stewart GD, Ross JA, Guttridge DC, Fearon KCH (2006) The molecular mechanisms of skeletal muscle wasting: implications for therapy. Surgeon 4:273–283 Available: http://www.ncbi.nlm.nih.gov/pubmed/17009546. Accessed: 2013 Dec 15. 17009546 10.1016/s1479-666x(06)80004-1

[12] Schiaffino S, Dyar KA, Ciciliot S, Blaauw B, Sandri M (2013) Mechanisms regulating skeletal muscle growth and atrophy. FEBS J 280:4294–4314 Available: http://www.ncbi.nlm.nih.gov/pubmed/23517348. Accessed: 2014 May 2. 23517348 10.1111/febs.12253

[13] Argilés JM, Busquets S, Toledo M, López-Soriano FJ (2009) The role of cytokines in cancer cachexia. Curr Opin Support Palliat Care 3:263–268 Available: http://www.ncbi.nlm.nih.gov/pubmed/19713854. Accessed: 2013 Nov 27. 19713854 10.1097/SPC.0b013e3283311d09

[14] Argilés JM, López-Soriano FJ, Busquets S (2012) Mechanisms and treatment of cancer cachexia. Nutr Metab Cardiovasc Dis 1–6 Available: http://www.ncbi.nlm.nih.gov/pubmed/22749678. Accessed: 2013 Nov 20. 10.1016/j.numecd.2012.04.011 22749678

[15] Macciò A, Madeddu C, Gramignano G, Mulas C, Floris C, et al (2012) A randomized phase III clinical trial of a combined treatment for cachexia in patients with gynecological cancers: evaluating the impact on metabolic and inflammatory profiles and quality of life. Gynecol Oncol 124:417–425 Available: http://www.ncbi.nlm.nih.gov/pubmed/22198049. Accessed: 2014 May 22. 22198049 10.1016/j.ygyno.2011.12.435

[16] Greig CA, Johns N, Gray C, Macdonald A, Stephens NA, et al (2014) Phase I/II trial of formoterol fumarate combined with megestrol acetate in cachectic patients with advanced malignancy. Support Care Cancer. doi:10.1007/s00520-013-2081-3 10.1007/s00520-013-2081-3 24389826

[17] Busquets S, Toledo M, Orpí M, Massa D, Porta M, et al (2012) Myostatin blockage using actRIIB antagonism in mice bearing the Lewis lung carcinoma results in the improvement of muscle wasting and physical performance. J Cachexia Sarcopenia Muscle 3:37–43 Available: http://www.pubmedcentral.nih.gov/articlerender.fcgi?artid=3302990&tool=pmcentrez&rendertype=abstract. Accessed: 2013 Nov 22. 22450815 10.1007/s13539-011-0049-z PMC3302990

[18] Zhou X, Wang JL, Lu J, Song Y, Kwak KS, et al (2010) Reversal of cancer cachexia and muscle wasting by ActRIIB antagonism leads to prolonged survival. Cell 142:531–543 Available: http://www.ncbi.nlm.nih.gov/pubmed/20723755. 20723755 10.1016/j.cell.2010.07.011

[19] Murphy KT, Chee A, Trieu J, Naim T, Lynch GS (2012) Importance of functional and metabolic impairments in the characterization of the C-26 murine model of cancer cachexia. Dis Model Mech 5:533–545 Available: http://www.pubmedcentral.nih.gov/articlerender.fcgi?artid=3380716&tool=pmcentrez&rendertype=abstract. Accessed: 2014 May 22. 22563056 10.1242/dmm.008839 PMC3380716

[20] Wilhelm SM, Carter C, Tang L, Wilkie D, McNabola A, et al (2004) BAY 43-9006 exhibits broad spectrum oral antitumor activity and targets the RAF/MEK/ERK pathway and receptor tyrosine kinases involved in tumor progression and angiogenesis. Cancer Res 64:7099–7109 Available: http://www.ncbi.nlm.nih.gov/pubmed/15466206. Accessed: 2013 Dec 15. 15466206 10.1158/0008-5472.CAN-04-1443

[21] Llovet JM, Ricci S, Mazzaferro V, Hilgard P, Gane E, et al (2008) Sorafenib in advanced hepatocellular carcinoma. N Engl J Med 359:378–390 Available: http://www.ncbi.nlm.nih.gov/pubmed/18650514. Accessed: 2014 Jan 22. 18650514 10.1056/NEJMoa0708857

[22] Kane RC, Farrell AT, Saber H, Tang S, Williams G, et al (2006) Sorafenib for the treatment of advanced renal cell carcinoma. Clin Cancer Res 12:7271–7278 Available: http://www.ncbi.nlm.nih.gov/pubmed/17189398. Accessed: 2014 Jan 22. 17189398 10.1158/1078-0432.CCR-06-1249

[23] Antoun S, Birdsell L, Sawyer MB, Venner P, Escudier B, et al (2010) Association of skeletal muscle wasting with treatment with sorafenib in patients with advanced renal cell carcinoma: results from a placebo-controlled study. J Clin Oncol 28:1054–1060 Available: http://www.ncbi.nlm.nih.gov/pubmed/20085939. Accessed: 2013 Nov 11. 20085939 10.1200/JCO.2009.24.9730

[24] Chai H, Luo AZ, Weerasinghe P, Brown RE (2010) Sorafenib downregulates ERK/Akt and STAT3 survival pathways and induces apoptosis in a human neuroblastoma cell line. Int J Clin Exp Pathol 3:408–415 Available: http://www.pubmedcentral.nih.gov/articlerender.fcgi?artid=2872747&tool=pmcentrez&rendertype=abstract. Accessed: 2013 Dec 15. 20490331 PMC2872747

[25] Liu L, Cao Y, Chen C, Zhang X, McNabola A, et al (2006) Sorafenib blocks the RAF/MEK/ERK pathway, inhibits tumor angiogenesis, and induces tumor cell apoptosis in hepatocellular carcinoma model PLC/PRF/5. Cancer Res 66:11851–11858 Available: http://www.ncbi.nlm.nih.gov/pubmed/17178882. Accessed: 2013 Dec 13. 17178882 10.1158/0008-5472.CAN-06-1377

[26] Yang F, Jove V, Buettner R, Xin H, Wu J, et al. (2012) Sorafenib inhibits endogenous and IL-6/S1P induced JAK2-STAT3 signaling in human neuroblastoma, associated with growth suppression and apoptosis: 534–541. 10.4161/cbt.19603 22406995

[27] Yang F, Brown C, Buettner R, Hedvat M, Starr R, et al (2010) Sorafenib induces growth arrest and apoptosis of human glioblastoma cells through the dephosphorylation of signal transducers and activators of transcription 3. Mol Cancer Ther 9:953–962 Available: http://www.pubmedcentral.nih.gov/articlerender.fcgi?artid=2852467&tool=pmcentrez&rendertype=abstract. Accessed: 2014 Oct 21. 20371721 10.1158/1535-7163.MCT-09-0947 PMC2852467

[28] Scott HR, McMillan DC, Crilly A, McArdle CS, Milroy R (1996) The relationship between weight loss and interleukin 6 in non-small-cell lung cancer. Br J Cancer 73:1560–1562 Available: http://www.pubmedcentral.nih.gov/articlerender.fcgi?artid=2074552&tool=pmcentrez&rendertype=abstract. Accessed: 2013 Dec 15. 8664130 10.1038/bjc.1996.294 PMC2074552

[29] Moses AGW, Maingay J, Sangster K, Fearon KCH, Ross JA (2009) Pro-inflammatory cytokine release by peripheral blood mononuclear cells from patients with advanced pancreatic cancer: relationship to acute phase response and survival. Oncol Rep 21:1091–1095 Available: http://www.ncbi.nlm.nih.gov/pubmed/19288013. Accessed: 2013 Dec 15. 19288013 10.3892/or_00000328

[30] Donati MB, Mussoni L, Poggi A, De Gaetano G, Garattini S (1978) Growth and metastasis of the Lewis lung carcinoma in mice defibrinated with batroxobin. Eur J Cancer 14:343–347 Available: http://www.sciencedirect.com/science/article/pii/0014296478902037. Accessed: 2014 Jan 9. 565716 10.1016/0014-2964(78)90203-7

[31] Fouladiun M, Körner U, Bosaeus I, Daneryd P, Hyltander A, et al (2005) Body composition and time course changes in regional distribution of fat and lean tissue in unselected cancer patients on palliative care–correlations with food intake, metabolism, exercise capacity, and hormones. Cancer 103:2189–2198 Available: http://www.ncbi.nlm.nih.gov/pubmed/15822132. Accessed: 2013 Dec 18. 15822132 10.1002/cncr.21013

[32] Das SK, Eder S, Schauer S, Diwoky C, Temmel H, et al (2011) Adipose triglyceride lipase contributes to cancer-associated cachexia. Science 333:233–238 Available: http://www.ncbi.nlm.nih.gov/pubmed/21680814. Accessed: 2013 Nov 14. 21680814 10.1126/science.1198973

[33] Hulmi JJ, Oliveira BM, Silvennoinen M, Hoogaars WMH, Pasternack A, et al (2013) Exercise restores decreased physical activity levels and increases markers of autophagy and oxidative capacity in myostatin/activin-blocked mdx mice. Am J Physiol Endocrinol Metab 305:E171–82 Available: http://www.ncbi.nlm.nih.gov/pubmed/23695214. Accessed: 2014 Jan 28. 23695214 10.1152/ajpendo.00065.2013

[34] Penna F, Costamagna D, Fanzani A, Bonelli G, Baccino FM, et al (2010) Muscle wasting and impaired myogenesis in tumor bearing mice are prevented by ERK inhibition. PLoS One 5:e13604 Available: http://www.pubmedcentral.nih.gov/articlerender.fcgi?artid=2965098&tool=pmcentrez&rendertype=abstract. Accessed: 2014 Jan 9. 21048967 10.1371/journal.pone.0013604 PMC2965098

[35] He WA, Berardi E, Cardillo VM, Acharyya S, Aulino P, et al (2013) NF-κB-mediated Pax7 dysregulation in the muscle microenvironment promotes cancer cachexia. J Clin Invest 123:4821–4835 Available: http://www.pubmedcentral.nih.gov/articlerender.fcgi?artid=3809785&tool=pmcentrez&rendertype=abstract. Accessed: 2014 Feb 5. 24084740 10.1172/JCI68523 PMC3809785

[36] Bonetto A, Aydogdu T, Kunzevitzky N, Guttridge DC, Khuri S, et al (2011) STAT3 activation in skeletal muscle links muscle wasting and the acute phase response in cancer cachexia. PLoS One 6:e22538 Available: http://www.pubmedcentral.nih.gov/articlerender.fcgi?artid=3140523&tool=pmcentrez&rendertype=abstract. Accessed: 2014 Jan 14. 21799891 10.1371/journal.pone.0022538 PMC3140523

[37] Penna F, Bonetto A, Muscaritoli M, Costamagna D, Minero VG, et al (2010) Muscle atrophy in experimental cancer cachexia: is the IGF-1 signaling pathway involved? Int J Cancer 127:1706–1717 Available: http://www.ncbi.nlm.nih.gov/pubmed/20039316. Accessed: 2014 May 28. 20039316 10.1002/ijc.25146

[38] Tierney MT, Aydogdu T, Sala D, Malecova B, Gatto S, et al (2014) STAT3 signaling controls satellite cell expansion and skeletal muscle repair. Nat Med 20:1182–1186 Available: http://www.ncbi.nlm.nih.gov/pubmed/25194572. Accessed: 2014 Sep 8. 25194572 10.1038/nm.3656 PMC4332844

[39] Price FD, von Maltzahn J, Bentzinger CF, Dumont NA, Yin H, et al (2014) Inhibition of JAK-STAT signaling stimulates adult satellite cell function. Nat Med 20:1174–1181 Available: http://www.ncbi.nlm.nih.gov/pubmed/25194569. Accessed: 2014 Sep 8. 25194569 10.1038/nm.3655 PMC4191983

[40] Prado CMM, Antoun S, Sawyer MB, Baracos VE (2011) Two faces of drug therapy in cancer: drug-related lean tissue loss and its adverse consequences to survival and toxicity. Curr Opin Clin Nutr Metab Care 14:250–254 Available: http://www.ncbi.nlm.nih.gov/pubmed/21415735. Accessed: 2013 Dec 5. 21415735 10.1097/MCO.0b013e3283455d45

