# Supplementary figures and images for: Distinct Behaviour of Sorafenib in Experimental Cachexia-Inducing Tumours: The Role of STAT3
Source: PLoS One. 2014 Dec 1;9(12):e113931. doi: 10.1371/journal.pone.0113931 (PMC4250056; doi:10.1371/journal.pone.0113931)

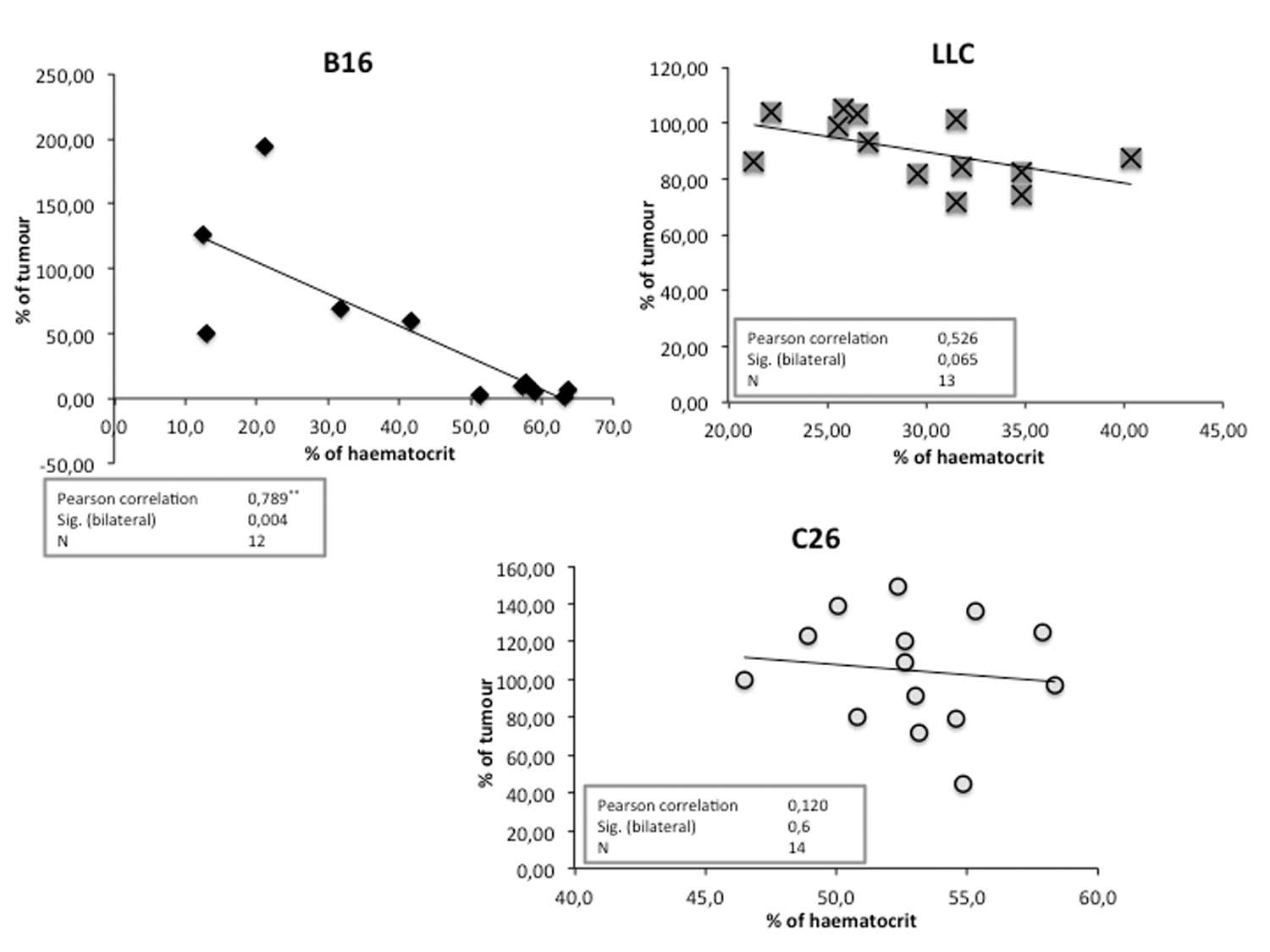

Supplement: Figure S1 — Correlation between tumour mass (expressed as % of the mean) and haematocrit in the three tumour models. Pearson analysis shows a significant correlation of tumour versus haematocrit in B16 (Pearson r = 0.789; p = 0.004, n = 12) and a tendency in LLC (Pearson r = 0.526; p = 0.065, n = 13). No correlation is present in C26 (Pearson r = 0.120; p = 0.6, n = 14). (TIF) [file pone.0113931.s001.tif]
